# Supplementary material for: Polyketide synthase gene clusters in the ericoid mycorrhizal fungus Oidiodendron maius: genome-wide prediction, expression profiling, and functional characterization of knockout mutants
Source: Front Plant Sci. 2026 Sep 16;17:1924430. doi: 10.3389/fpls.2026.1924430 (PMC13623903; doi:10.3389/fpls.2026.1924430)
Supplement: Supplementary file 1 [file SupplementaryFile1.zip › Supplementary Materials/Supplementary Folder 1.zip!/Oidma1_AssemblyScaffolds_Repeatmasked/knownclusterblast/cluster9/jgi.p_Oidma1_107312_mibig_hits.html]

| MIBiG Protein | Description | MIBiG Cluster | MiBiG Product | % ID | % Coverage | BLAST Score | E-value |
| --- | --- | --- | --- | --- | --- | --- | --- |
| ABW71833.1 | putative\_aldehyde\_dehydrogease | BGC0000303 | Anthramycin\_biosynthetic\_gene\_cluster | 33 | 98.9539748954 | 234 | 2.9e-61 |
| CAQ52616.1 | benzaldehyde\_dehydrogenase | BGC0001066 | Kendomycin\_biosynthetic\_gene\_cluster | 33 | 96.8619246862 | 218 | 2.1e-56 |
| ALI92651.1 | CitD\_dehydrogenase | BGC0001338 | Citrinin\_biosynthetic\_gene\_cluster | 32 | 97.6987447699 | 214 | 3.1e-55 |
| WP\_030845402.1 | aldehyde\_dehydrogenase | BGC0001368 | Tambromycin\_biosynthetic\_gene\_cluster | 33 | 97.0711297071 | 205 | 1.4e-52 |
| BAP16694.1 | aldehyde\_dehydrogenase | BGC0000376 | JBIR-34,\_JBIR-35\_biosynthetic\_gene\_cluster | 32 | 95.3974895397 | 205 | 1.9e-52 |
| BAK64640.1 | putative\_aldehyde\_dehydrogenase | BGC0000135 | Reveromycin\_biosynthetic\_gene\_cluster | 31 | 99.5815899582 | 198 | 1.3e-50 |
| CAJ14050.1 | putative\_aldehyde\_dehydrogenase | BGC0000406 | Phosphinothricin\_biosynthetic\_gene\_cluster | 33 | 95.6066945607 | 194 | 2.5e-49 |
| CAE45692.1 | hypothetical\_protein | BGC0000031 | Borrelidin\_biosynthetic\_gene\_cluster | 30 | 93.0962343096 | 171 | 1.8e-42 |
| EDY47105.1 | aldehyde\_dehydrogenase | BGC0000319 | Cephamycin\_C\_biosynthetic\_gene\_cluster | 28 | 96.0251046025 | 163 | 8.1e-40 |
| CAJ88625.1 | putative\_aldehyde\_dehydrogenase | BGC0000327 | Congocidine\_biosynthetic\_gene\_cluster | 30 | 94.9790794979 | 152 | 1.1e-36 |
